# Predictors of recurrence and long-term patient reported outcomes following surgical repair of anal fistula, a retrospective analysis

**DOI:** 10.1007/s00384-024-04602-1

**Published:** 2024-03-11

**Authors:** Sidrah Khan, Rebecca Kotcher, Paul Herman, Li Wang, Robert Tessler, Kellie Cunningham, James Celebrezze, David Medich, Jennifer Holder-Murray

**Affiliations:** 1 Department of Surgery, University of Pittsburgh Medical Center, Kaufmann Medical Building, Suite 603, 3471 Fifth Avenue, Pittsburgh, PA 15213 USA; 2 Department of Surgery, University of Washington Medical Center, Seattle, WA USA; 3 Division of Colorectal Surgery, Department of Surgery, University of Pittsburgh Medical Center, Kaufmann Medical Building, Suite 603, 3471 Fifth Avenue, Pittsburgh, PA 15213 USA; 4 Clinical and Translational Science Institute, University of Pittsburgh Medical Center, Kaufmann Medical Building, Suite 603, 3471 Fifth Avenue, Pittsburgh, PA 15213 USA

**Keywords:** Anal, Fistula, LIFT, Sphincterotomy, Incontinence

## Abstract

**Purpose:**

Surgery for anal fistulas can result in devastating complications, including reoperations and fecal incontinence. There is limited contemporary evidence comparing outcomes since the adoption of the ligation of intersphincteric fistula tract procedure into mainstream practice. The purpose of this study is to compare recurrence rates and long-term outcomes of anal fistula following repair.

**Methods:**

Data was collected from the electronic medical records or patient reported outcomes from patients aged 18 or older with a primary or recurrent cryptoglandular anal fistula. Primary outcome was recurrence defined as the identification of at least one fistula os or a high clinical suspicion of anal fistula. Secondary outcomes included fecal incontinence and postoperative quality of life.

**Results:**

A total of 171 patients underwent definitive surgical repairs for their anal fistula. So 66.5% had a simple fistula, and 33.5% had a complex fistula. Of the 171 patients, 12.5% had a recurrence. The recurrence rates were 5.9% for simple fistula and 25.4% for complex fistula. Predictors of recurrence included diabetes mellitus, history of anorectal abscess, complex fistula, and sphincter sparing surgery. LIFT or plug/biologic procedures were both associated with a 50% or greater recurrence rate. No significant differences were found in fecal incontinence or associated quality of life between sphincter sparing or non-sphincter sparing surgical resections.

**Conclusion:**

The study provides insights into the long-term outcomes of surgical repair for anal fistula. We demonstrate that sphincter sparing operations are associated with increased recurrence, meanwhile, non-sphincter sparing surgeries did not increase the risk of fecal incontinence or worsen quality of life.

## Introduction

Cryptoglandular anal fistulas are a common anorectal pathology that can have vexing clinical courses with variable healing rates [1, 2]. Even when complete healing occurs, incontinence can ensue [1, 2]. The prevalence varies widely depending on geographic location and ethnicity, with an estimated incidence of 1 per 10,000 people in developed countries and up to 9 per 1000 in developing countries [3, 4]. While the exact cause of anal fistula is not always clear, it is often associated with infection in the anal glands or a history of chronic constipation or fecal impaction [4]. Some case reports also link anal fistula formation with prolonged periods of sitting on the toilet and even pregnancy [5].

Treatment for anal fistulas can be complex. The initial treatment aims to drain any associated infection and achieve source control [6, 7]. Definitive management involves eradicating the fistula while preserving anal sphincter function and avoiding recurrence of the disease. The methods of treating anal fistulas vary significantly depending on the type of fistula. Parks et al. were the first to categorize types of fistulas based on their relation to the sphincter muscle [8]. Based on the type of anal fistula, management options include non-sphincter sparing surgeries (fistulotomy, fistulectomy, or cutting seton) versus sphincter sparing surgeries (ligation of intersphincteric tract (LIFT), endoanal advancement flap, or biological graft plug).

Anal fistula surgery can result in devastating complications, the most serious of which are recurrence of disease necessitating reoperations or fecal incontinence. Studies show the recurrence rates for anal fistula surgery range between 5 and 50%, with up to a 40% rate of fecal incontinence leading to significantly diminished quality of life [9, 10]. There are a variety of patient, fistula, and surgery-related risk factors that can influence anal fistula as well as their outcomes from surgery. These include age, race/ethnicity, duration of symptoms, comorbidities, type and location of fistula, and surgical technique. There is limited contemporary evidence comparing the outcomes of anal fistula surgery, especially since the adoption of the LIFT procedure into mainstream practice. In this study, we aimed to compare the recurrence rates of cryptoglandular anal fistula following definitive repair as well as to characterize the long-term functional outcomes based on fistula classification and surgical repair type in a real-world setting.

## Materials and methods

### Patients

This is a retrospective analysis of prospectively collected data. All patients aged 18 or older with a primary or recurrent cryptoglandular anal fistula between 2011 and 2019 who underwent surgical repair at one of two academic medical centers within a single healthcare system were initially included in this study. Additional inclusion criteria included identification of both external and internal os and a definitive repair operation. Patients were excluded if there was missing data of type of fistula or surgery, if the fistula was determined to be non-cryptoglandular in origin (i.e., Crohn’s disease, HIV, malignant neoplasm, obstetrical trauma, or other organ involvement included colovesical, diverticular, and rectovaginal), if the only management was a non-definitive repair such as a draining seton, or if no fistula was identified. All surgeries were performed by colorectal surgeons within the health system.

### Data collection

This study was performed under the approval of the University of Pittsburgh institutional review board, protocol STUDY19070137. Intraoperative findings, surgical repair, and outcome data were collected from the electronic medical record or patient reported outcomes. Anal fistulas were classified as simple or complex fistula. Simple fistulas were comprised of intersphincteric, or low/very low transsphincteric fistulas. Complex fistulas included mid/high transsphincteric and suprasphincteric. The primary outcome was recurrence of fistula by clinical exam finding. Fistula recurrence was defined as the identification of at least one fistula os (internal or external) or high clinical suspicion of anal fistula based on clinical exam with associated regular drainage and pain. Presence or persistence of the anal fistula at 6 months from the definitive repair surgery was noted to be failure of healing. If the fistula had healed but was identified more than 6 months after the initial surgery, it was determined to be a recurrent fistula. If a fistula was found in an unrelated location from the treated fistula, it was categorized as its own entity instead of a recurrence.

Secondary outcomes included fecal incontinence defined by the Wexner score and postoperative quality of life defined by the fecal incontinence quality of life (FIQL) scale. The Wexner score, also known as the Cleveland Clinic Florida Fecal Incontinence Severity Scoring System (CCFIS), is a classification system used to categorize the severity of fecal incontinence [11]. We utilized the FIQL scale, a Likert-type questionnaire that evaluates the negative impact fecal incontinence has on quality of life [12]. It is divided into 4 domains: lifestyle, coping/behavior, depression/self-perception, and embarrassment [12]. Patients were consented for phone surveys prior to or after their surgery. These scripted phone surveys were performed by two interviewers between February 2021 and August 2021 and included questions comprising the Wexner score and the FIQL scale.

### Data analysis

Data were presented as mean with standard deviation (SD) or median with interquartile range (IQR) for continuous variables and frequency with percentage for categorical variables. Univariate analysis was performed using Chi-square test or Fisher’s exact test for categorical variables and Mann Whitney *U* test for continuous variables. Univariate odds ratios and 95% confidence intervals were reported for fistula recurrence using logistic regression. Due to the small number of recurrences observed in our patients, no multivariable analysis was performed. A *p* value < 0.05 was considered statistically significant. SPSS version 27 was used for statistical analysis (Armonk, NY).

## Results

**Fig. 1:**
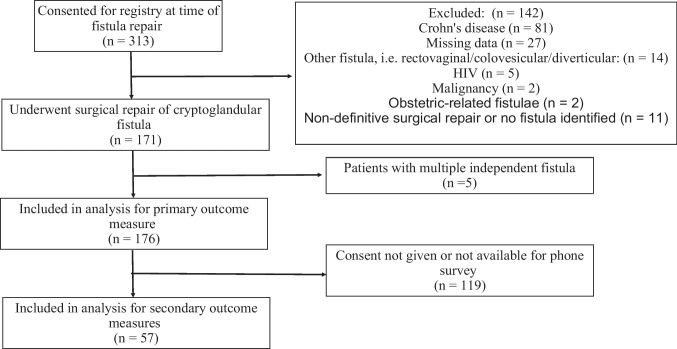
Patient flowchart

A total of 312 patients underwent surgery for anal fistula between 2011 and 2019 (Fig. 1). Of these, 142 patients were excluded for fistula disease of non-cryptoglandular pathology, non-definitive treatment of their fistula, or missing data (Fig. 1). The remaining 171 patients underwent definitive surgical repairs for the anal fistula. Of these, 5 patients had 2 fistulas in distinct locations and were counted as separate entities. This resulted in a total of 176 surgical repairs being included in the analysis for the primary outcome of recurrence (Fig. 1). Of the 171 patients, 108 (63.2%) were males with a median age of 50 (IQR 40–61) and the median BMI of 31 (IQR 26–35) (Table 1). Drainage was the primary complaint upon presentation for the majority of patients, *n* = 137 (80.1%). A total of 114 patients (65.5%) had a prior non-definitive surgical management for their perianal fistula which included exam under anesthesia with or without seton placement and/or subcutaneous fistulotomy. Only 2 (*n* = 11.6%) patients had a prior recurrence. Twenty-three patients (13.4%) had MRIs of their pelvis as a workup prior to surgery. The remaining demographics and patient characteristics are listed in Table 1.

**Table 1:** Baseline characteristics

**Characteristic**	***n*** **(%)**
**Sex**	
Male	108 (63.5)
**Age (y, at the time of first surgery), median (IQR)**	50 (40–61)
** < 45**	62 (35)
** > 45**	114 (65)
**Race**	
White	151 (88.8)
**Presenting symptoms**	
Drainage	137 (80.1)
Pain	78 (45.6)
**Previous history of anorectal pathologies**	
Anorectal abscess	107 (62.6)
Hemorrhoids	23 (13.5)
Fecal incontinence	1 (0.6)
**Prior surgery for anal fistula**	
None	45 (26.3)
Non-definitive repair	114 (66.6)
Definitive repair	12 (7.0)
**Other prior anorectal surgery**	
Hemorrhoidectomy	13 (7.6)
Partial lateral internal sphincterotomy	6 (3.5)
**BMI (kg/m**^**2**^**), median (IQR)**	31 (26–35)
**Diabetes mellitus**	13 (7.6)

Fistulas were classified into two broad categories, simple (*n* = 117, 66.5%) or complex (*n* = 59, 33.5%). Simple fistulas were comprised of intersphincteric, or low/very low transsphincteric fistulas. Complex fistulas included mid/high transsphincteric and suprasphincteric. The distribution across subgroups was as follows: low/very low transsphincteric fistulas (*n* = 75, 42.6%), intersphincteric fistulas (*n* = 42, 23.8%), mid/high transsphincteric fistulas (*n* = 58, 32.9%), intersphincteric fistulas (*n* = 42, 23.8%), and suprasphincteric fistulas (*n* = 1, 0.1%). For simple fistulas, fistulotomies were the most common surgical intervention performed (*n* = 107, 91.5%), followed by LIFT procedures (*n* = 4, 3.4%), cutting setons (*n* = 4, 3.4%), fistulectomy (*n* = 1, 0.1%), and endoanal advancement flaps (*n* = 1, 0.1%). For complex fistulas, the predominant surgical intervention was cutting setons (*n* = 29, 49.2%) and then LIFT (*n* = 16, 27.1%) and plug or biologic graft (*n* = 10, 16.9%) (Table 2).

**Table 2:** Surgery type according to fistula classification

**Fistula classification**		**Non-sphincter-sparing surgery**			**Sphincter-sparing surgery**			**Total** ***n*** **(%)**
		**Fistulotomy** ***n* (%)**	**Fistulectomy** ***n* (%)**	**Cutting seton** ***n* (%)**	**Plug or biologic graft *n* (%)**	**Endoanal advancement flap *n* (%)**	**LIFT** ***n* (%)**	
**Simple**	**Intersphincteric**	39 (93)	0 (0)	1 (2.3)	0 (0)	1 (2.3)	1 (2.3)	42 (23.8)
	Low transsphincteric/very low transsphincteric *	68 (90.6)	1 (1.6)	3 (4.8)	0 (0)	0 (0)	3 (4.8)	75 (35.7)
**Complex**	Mid/high transsphincteric*	4 (7)	0 (0)	29 (50)	9 (15.5)	0 (0)	16 (27.5)	58 (32.9)
	**Suprasphincteric**	0 (0)	0 (0)	0	1 (100)	0 (0)	0 (0)	1 (0.08)
**Total**		111 (63)	1 (0.5)	33 (19)	10 (5.6)	1 (0.5)	20 (11.4)	176 (100)

*Low, mid, and high transsphincteric involve < 33%, 33–50%, and > 50% of external anal sphincter muscle, respectively

*LIFT* ligation of intersphincteric fistula tract

Of the 171 patients who underwent 176 surgical procedures for their anal fistulas, 22 (12.5%) had recurrence of their disease. Seven (5.9%) of the patients with simple fistulas had a recurrence compared to 15 (25.4%) with complex fistulas. All recurrences in patients with complex fistulas were in those with mid/high transsphincteric fistulas. Among these patients, the highest recurrence rates were seen in those who underwent a plug or biologic graft placement (*n* = 5, 55.6%). Among these patients, those who underwent a LIFT procedure had a 50% recurrence rate (*n* = 8). However, patients with a mid/high transsphincteric fistula who underwent either a cutting seton placement, fistulotomy, or fistulectomy had recurrence rates of 6.9% (*n* = 2), 0% (*n* = 0), and 0% (*n* = 0), respectively. In patients with simple fistulas, fistulotomies were associated with a 10.2% (*n* = 7) recurrence rate, whereas LIFT and endoanal advancement flap procedures were associated with recurrence rates of 25% (*n* = 1) and 100% (*n* = 1), respectively (Table 3). Of the preoperative and operative variables, predictors of recurrence included diabetes mellitus (OR 4.74; CI 1.42–15.79; *p* = *0.018*), history of anorectal abscess (OR 3.20; CI 1.03–9.90; *p* = *0.035*), complex fistula (OR 0.19; CI 0.07–0.49; *p* < 0.001), and sphincter sparing surgery (OR 0.05; CI (0.02–0.15); *p* < *0.001*) (Table 4).

**Table 3:** Recurrences according to fistula classification and surgery type

**Fistula classification**		**Non-sphincter-sparing surgery**			**Sphincter-sparing surgery**			**Total**
		**Fistulotomy** ***n* (%)**	**Fistulectomy** ***n* (%)**	**Cutting seton** ***n* (%)**	**Plug or biologic** **graft *n* (%)**	**Endoanal** **advancement** **flap *n* (%)**	**LIFT** ***n* (%)**	
**Simple**	**Intersphincteric**	0 (0)	0 (0)	0 (0)	0 (0)	1 (100)	0 (0)	1 (2.3)
	Low transsphincteric/very low transsphincteric *	7 (10.2)	0 (0)	0 (0)	00 (0)	0 (0)	1 (33.3)	8 (10.6)
**Complex**	Mid/high transsphincteric*	0 (0)	0 (0)	2 (6.9)	5 (55.6)	0 (0)	8 (50)	15 (25.8)
	**Suprasphincteric**	0 (0)	0 (0)	0 (0)	0 (0)	0 (0)	0 (0)	0 (0)
**Total**		7 (6.3)	0 (0)	2 (6.1)	5 (50)	1 (100)	9 (45)	22 (12.5)

*Low, mid, and high transsphincteric involve < 33%, 33–50%, and > 50% of external anal sphincter muscle, respectively

*LIFT* ligation of intersphincteric fistula tract

**Table 4:** Predictors of recurrence

**Characteristic**	**Recurrence rate (%)**	**Odds ratio (95% CI)**	***p*** **Value**
**Age**		0.76 (0.30–1.89)	0.551
≤ 45 years	14.5		
> 45 years	11.2		
**Sex**		0.98 (0.39–2.49)	0.971
Female	12.5		
Male	12.4		
**Race**		0.84 (0.23–3.14)	0.732
White	12.3		
Non-white	14.3		
**BMI (kg/m**^**2**^**)**		0.95 (0.26–3.52)	1.00
< 25	12.0		
≥ 25	11.5		
**History of anorectal abscess**		3.20 (1.03–9.90)	0.035
No	5.9		
Yes	16.7		
**History diabetes**		4.74 (1.42–15.79)	0.018
No	10.4		
Yes	35.7		
**History smoking**		0.59 (0.23–1.57)	0.266
No	15.2		
Yes	9.5		
**Prior incision and drainage**		0.69 (0.08–5.63)	0.725
No	1.4		
Yes	19.6		
**Fistula classification**		0.19 (0.07–0.49)	< 0.001
Simple	6.0		
Complex	25.4		
**Surgery type**		0.05 (0.02–0.15)	< 0.001
Sphincter-sparing	48.4		
Non-sphincter-sparing	4.8		

*CI* confidence interval

This study also assessed the fecal incontinence rates and associated quality of life following definitive anal fistula repair. Fifty-seven of the 171 patients (33%) consented to receiving a follow-up phone call and responded to the scripted questionnaire with a median follow-up at the time of the phone survey of 6.7 years (1.4–9.4 years). The mean postoperative Wexner score for simple fistula was 1.2 ± 2.11 compared to 3.0 ± 3.56 for complex fistula (*p* = *0.008*) (Table 5). No significant differences were found in fecal incontinence by the Wexner score between patients who underwent sphincter sparing or non-sphincter sparing surgical resections (*p* = *0.219*) (Table 5). Preoperative variables associated with worsened fecal incontinence included age greater than 45 (*p* = *0.041*) and history of smoking (*p* = *0.030*). When looking at the effect of postoperative fecal incontinence on the quality of life, the only factor associated with worse quality of life was age greater than 45 (*p* = *0.008*). Gender, fistula classification, nor type of surgery were associated with a difference in the FIQL score.

**Table 5:** Predictors of Wexner and FIQL score

**Characteristic**	**Wexner score, mean (SD)**	***p*** **Value**	**FIQOL score, mean (SD)**	***p*** **Value**
**Age**		0.041		0.008
≤ 45 years	0.67 (1.23)		16.01 (0.11)	
> 45 years	1.23 (3.16)		15.16 (1.99)	
**Sex**		0.111		0.562
Female	1.15 (1.95)		15.56 (1.19)	
Male	2.36 (3.23)		15.24 (2.05)	
**Race**		0.414		0.738
White	2.00 (2.96)		15.33 (1.82)	
Non-white	0.75 (1.50)		15.95 (0.082)	
**BMI (kg/m**^**2**^**)**		0.952		0.829
< 25	1.55 (2.00)		15.77 (0.39)	
≥ 25	2.00 (3.03)		15.27 (1.94)	
**History diabetes**		0.135		0.149
No	1.68 (2.42)		15.56 (1.29)	
Yes	5.67 (7.32)		12.51 (4.86)	
**History smoking**		0.030		0.220
No	1.37 (2.69)		15.45 (1.83)	
Yes	2.86 (3.02)		15.22 (1.68)	
**Prior incision and drainage**		0.671		0.717
No	1.94 (2.91)		15.45 (1.69)	
Yes	1.40 (2.60)		14.51 (2.42)	
**Fistula classification**		0.008		0.102
Simple	1.20 (2.11)		15.64 (1.23)	
Complex	3.00 (3.56)		14.86 (2.42)	
**Surgery type**		0.219		0.130
Sphincter-sparing	3.40 (4.52)		14.03 (3.34)	
Non-sphincter-sparing	1.57 (2.32)		15.64 (1.15)	

*SD* standard deviation

## Discussion

Although the true prevalence of anal fistulas is unknown, they are a common and potentially devastating pathology. When left untreated, fistulas can lead to chronic infections, abscesses, and persistent perianal drainage. The definitive repair of anal fistulas can be challenging and often requires a tailored approach based on the individual fistula’s anatomy. Since the addition of LIFT procedures to the colorectal surgeon’s armamentarium for treatment of anal fistula, little data exists comparing outcomes. In this descriptive study, we assessed the long-term outcomes of surgical repair in a large consecutive series of patients with simple or complex fistulas of cryptoglandular origin. Our study demonstrates that mid/high transsphincteric fistulas are associated with the highest recurrence rates. More importantly, both LIFT and plug/biologic procedures were associated with a 50% or greater recurrence in mid/high transsphincteric anal fistulas. Interestingly, sphincter sparing surgeries were not associated with any improvement in postoperative fecal incontinence or fecal incontinence associated quality of life when compared to non-sphincter sparing operations.

Among the preoperative variables, our data show diabetes mellitus to be associated with increased risk of recurrence. Although this seems intuitive as elevated blood glucoses can delay wound healing in many ways, published studies have shown both diabetics and non-diabetics to have an increased risk of recurrence [13]. A clear understanding of why non-diabetics in some studies have been shown to have equal or higher rates of recurrences is lacking [13]. Other preoperative variables in our study that are associated with higher rates of recurrences are history of anorectal abscesses and subsequent drainage procedures. Published literature support these findings, demonstrating that the percentage of repeat anorectal abscesses and anal fistula has been estimated to fall between 25 to 50%. Inadequate drainage and abscesses are the primary technical reasons cited [14, 15].

Most studies assessing the anatomic or surgical variables associated with recurrence have shown conflicting results [9, 14–17]. Our findings reveal that the overall recurrence rate following definitive anal fistula repair is 12.5% and recurrence varies based on the complexity of the anal fistula. Two studies have reported recurrence rates lower than ours with rates between 7 and 8% [9, 18]. Garcia-Aguilar et al. investigated 375 patients who underwent surgical interventions for simple and complex anal fistula and reported a recurrence rate of 8% [9]. The procedures studied in this paper included fistulotomy, seton placement, and endorectal advancement flaps. Jordán et al. at investigated 279 patients with anal fistula and reported a recurrence rate of 7.2% [18]. Of their patient cohort, 42.7% were categorized as having complex fistula with surgical procedures including fistulotomies, fistulectomies, and endorectal advancement flaps [18]. Both of these studies lacked inclusion of procedures such as LIFT or plug/biologic graft placement and had a very short follow-up of approximately 4 months. In contrast, a third retrospective review by Abbas et al*.* investigated the outcomes of anal fistula surgery in 179 patients and demonstrated an operative failure rate of 15.6% [19]. Even though this study assesses a variety of surgical procedures including fistulotomies, endorectal advancement flaps, or plug/biologic graft placement, the variability in definitions of outcomes from ours makes it challenging to interpret as recurrence/persistence of disease was assessed at a short interval with a median follow-up of less than 2 months [19]. The short follow-up in these studies likely skews the reported healing rates.

Our data demonstrate that while intersphincteric and low/very low transsphincteric fistulas are the most prevalent types, mid/high transsphincteric fistulas have the highest rates of recurrence. Furthermore, undergoing a LIFT or plug/biologic procedure increases the risk of recurrence to greater than 50%, meanwhile undergoing a cutting seton placement leads to very low recurrence rates, around 7%. Published studies looking at the outcomes after LIFT procedures show variable rates of recurrence. One retrospective study assessed 45 patients who underwent LIFT procedures and reported a recurrence rate of 40% [20]. In their patient cohort, majority (84%) had complex anal fistulas. Interestingly, they also reported LIFT procedures to be associated with a 75% reoperation rate [20]. Even though the recurrence rate reported here is comparable to that demonstrated from our data, it is challenging to interpret because of the limited sample sizes. Furthermore, our study did not look at the reoperation rates after LIFT procedures given the small sample size. Another cohort performed a randomized controlled trial which compared 118 patients who underwent LIFT procedures with 117 patients who had LIFT and plug. After a 6-month follow-up, they reported LIFT procedures to have an 83.9% healing rate compared to a 94% healing rate in LIFT+plugs. Furthermore, they reported no recurrences, but unfortunately with only a short-term follow-up of 6 months [21]. The data from this study are difficult to compare to ours as the follow-up period and definition of recurrence differs from that in our study. Unfortunately, there is no universal definition of recurrence. Some studies report recurrence to be reemergence of disease after complete healing, while others equate it to non-healing. The time point at which recurrence is measured also varies between studies. Additionally, data on LIFT procedures are inconsistent in terms of inclusion criteria, surgical technique, and often lack reproducibility. One systematic review looked at 26 studies that included randomized control trials and cohort/case series [22]. They reported seven technical variations as well as healing rates to vary from 47 to 95% [22]. This variability presents significant challenges in comprehending the true outcomes of LIFT procedures. When considering plug/biologic graft placement, a review of 64 articles including multiple randomized clinical trials reported a healing rate of 50–60% in complex anal fistulas [23]. They reported these outcomes to be similar to that seen with LIFT procedures and recurrence rates to be similar to endorectal advancement flaps [24]. This study concluded that plugs/biologics are a good option with a near 50% success rate and low complication rate; however, most centers do not have nearly such high success and utilize this technique sparingly. Another study evaluated 21 patients who underwent anal fistula plugs for a high inter- or transsphincteric fistula. They reported 76.2% healing rate with a median follow-up of 20.9 months. Although they report very encouraging results, rates of recurrence after complete healing were not discussed [25]. Our data show the lowest recurrence rates to be associated with cutting setons. Although cutting setons have become less favorable given the potential risks of fecal incontinence, the outcomes from our data are positive. A large number of patients who underwent cutting setons in this study are also in part due to the time period in which this data were collected, where LIFT procedures were newly being introduced.

Fecal incontinence can have a dramatically negative impact on a person’s quality of life, as it can lead to embarrassment, social isolation, and decreased self-esteem. One potential benefit of sphincter sparing procedures is that they are generally believed to carry a lower risk of fecal incontinence, although evidence on this point is conflicting. We utilized standardized measurements of fecal incontinence and its associated quality of life (Wexner and FIQL scores). Of the preoperative variables, age and history of smoking were associated with increased rates of fecal incontinence. Only age was linked to a worsened quality of life. Interestingly, even though our data show that complex fistulas are linked to higher rates of fecal incontinence, preservation of the sphincter is not, as we report similar outcomes in terms of fecal incontinence and quality of life in patients who underwent cutting setons compared to sphincter preserving procedures. Overall, there were no associations between the types of fistulas or surgical repair with worsened quality of life. Some studies do report low rates of fecal incontinence (5%), while others depict rates of incontinence at around 45% [9, 24–27]. The inconsistencies in these studies are due to a multitude of factors, including whether the studies accounted for preoperative levels of fecal incontinence and the variability in measurement of incontinence and quality of life.

Our study has several limitations. First, this is a retrospective study of a single institutional experience, so whether it can be applied to other health systems and patient populations is debatable. Furthermore, although we have a large cohort of patients who underwent surgical interventions for anal fistula, there is heterogeneity in the procedures in that there are a large number of cutting setons performed and low number of LIFT procedures. Such a high rate of cutting setons is not in line with the present national trend of management, and such low numbers of LIFT procedures brings difficulty in interpreting results. However, we offer a real-world perspective and comparison of types of surgeries such as LIFT and cutting setons, which should be further studied. In addition, because the procedures were not chosen at random, there is an inherent bias to the data. Nonetheless, we do have a long-term follow-up with a median of more than 6 years. Another shortcoming is that in a majority of our patients, physical exam is the only tool utilized to establish recurrence without adding in other manners of surveillance such as MRI. While this is a limitation of this retrospective study, it represents real world practice. Our study had a low response rate of 33% to examine long-term functional outcomes, which can lead to its own biases. Lastly, this study lacks an assessment of preoperative fecal incontinence for comparison to the postoperative fecal incontinence and its associated quality of life. It would be beneficial to carry out a randomized controlled trial with different surgical techniques in a patient cohort with a variety of fistula types and with a long-term follow-up.

## Conclusion

We demonstrate that following definitive surgical repair, simple fistulas are associated with a 5.9% recurrence rate, whereas complex fistulas have a much higher recurrence rate of 25.4%. Procedures such as plug/biologic or LIFT, which spare the sphincter, are associated with the highest recurrence rates of over 50%. Our data indicate that non-sphincter sparing approaches to fistula surgery are associated with lower rates of recurrence and do not lead to increased likelihoods of postoperative fecal incontinence. Further randomized studies with long-term follow-up would be beneficial to identify optimal surgical technique based on fistula type.

## Data Availability

No datasets were generated or analyzed during the current study.
